# The Corrosion Resistance of Reinforced Magnesium Phosphate Cement Reactive Powder Concrete

**DOI:** 10.3390/ma15165692

**Published:** 2022-08-18

**Authors:** Zhiqiang Xu, Peng Cao, Di Wang, Hui Wang

**Affiliations:** 1 School of Chemical and Mechanical Engineering, Liaodong University, Dandong 118001, China; 2 College of Architecture and Civil Engineering, Beijing University of Technology, Beijing 100124, China; 3 School of Civil and Environmental Engineering, Ningbo University, Ningbo 315000, China

**Keywords:** magnesium phosphate, reactive powder concrete, NaCl freeze-thaw cycles, NaCl dry-wet alternations, corrosion resistance

## Abstract

Magnesium phosphate cement-based reactive powder concrete (MPC-RPC) is a cement-based material with early strength, high strength and excellent durability. The slump flow and setting time of steel fibers reinforced MPC-RPC are investigated. Meanwhile, the flexural strength, the compressive strength, the ultrasonic velocity and the electrical resistivity of specimens cured for 3 h, 1 day, 3 days and 28 days are determined. Moreover, the corresponding corrosion resistance reinforced MPC-RPC exposing to NaCl freeze-thaw (F-T) cycles and dry-wet (D-W) alternations is researched. In this study, the steel fibers used are 0%, 0.5%, 1.0%, 1.5%, 2.0%, 2.5% and 3.0% by the volume of MPC-RPC. The corrosion of the inner reinforcement is reflected using the mass loss, electrical resistivity, ultrasonic velocity, and the AC impedance spectrum. Researching findings show that the steel fibers lead to decreasing the slump flow and setting time. The flexural strength, the compressive strength and ultrasonic velocity of MPC-RPC cured for 3 h are higher than 45% of the MPC-RPC cured for 28 days. Moreover, when the MPC-RPC is cured for 7 days, the flexural strength, the compressive strength and ultrasonic velocity of MPC-RPC are higher than 85% of the specimens cured for 28 days. The electrical resistance decreases in a quadratic function as the volume ratio of steel fibers increases. The corrosion resistance of the internal reinforcement can be improved by adding steel fibers at appropriate dosages. The reinforcement inner MPC-RPC corrodes more seriously under the NaCl D-W alternations than NaCl F-T cycles.

## 1. Introduction

Cement concrete is a type of material with high mechanical properties and good durability, which has been used in civil engineering industry for many years [1,2]. Recently, the frequent construction of large marine concrete structures provides a platform for the development and application of cement concrete [3]. The sea crossing bridge is the hub of coastal cities, which usually encounters complex application environment similar to scouring, dry-wet (D-W) alternations and freeze-thaw (F-T) cycles of seawater [4,5,6]. The expansion joint anchorage zone of sea crossing bridge encounters the action of complex loads. Due to the erosion by sea water and the complex loads, the cement concrete of the anchorage zone of bridge expansion joint materials is frequently damaged.

Sulphoaluminate cement matrix, the Portland cement matrix, the magnesium phosphate cement matrix and compound cement matrix are usually applied in the rapid repairing of cement concrete constructions [7,8,9,10,11]. Sulphoaluminate cement-based materials have been proved to possess quite high mechanical strength at early curing age (less than 1 d) [3]. However, the mechanical strength at later curing age is lower than that of Portland cement concrete. Meanwhile, the durability of magnesium phosphate cement matrix in marine environment is inferior [12]. Portland cement-based materials with early strength agent and the compound cement matrix show better later mechanical strength and durability than the cement concrete with only sulphoaluminate cement. This kind of concrete is unable to open to traffic until the curing age reaches 3 days.

Magnesium phosphate cement matrix is generally manufactured by combining the magnesium oxide and potassium dihydrogen phosphate [13,14]. This kind of cement matrix is simple to prepare and has good mechanical properties and durability [15,16,17,18]. Moreover, the magnesium phosphate cement matrix shows excellent bonding performance and impermeability. Du’s research pointed out that the addition of MgCl_2_ can be used for delaying the setting of magnesium phosphate cement and reducing the corresponding early cracks and the drying shrinkage rate [11]. Zhang et al. [19] found that the magnesium phosphate cement shows good resistance to NaCl F-T cycles and acid and alkali resistance. However, plain concrete is rarely used in real engineering.

When cement concrete is used in practical engineering, a certain amount of steel bars needs to be prepared. The steel bars’ inner cement concrete is prone to corrosion during service, due to the fact that the iron loses electrons to form iron compounds, and the corrosion occurs [20,21,22]. When the steel bars reinforced cement concrete is applied in the marine environment, the inner steel bars usually corrode seriously [23,24,25,26,27]. This is because chloride ions corrode the passive film of the steel bars, resulting in the increased corrosion [28,29]. The expansion joint is widely used mechanical connecting device with complex external load. The magnesium phosphate cement-based materials are often applied in the replacement of concrete in anchorage zone of bridge expansion joint. A certain amount of reinforcement is usually prepared in the anchorage zone of bridge expansion joint. Therefore, when the magnesium phosphate cement matrix is used for replacing the anchorage zone, the corrosion of the inner steel bars should be considered. However, little research about this has been reported.

As an ultra-dense material, reactive powder concrete (RPC) has been developed in 1990s. RPC usually consists of a large amount of mineral admixtures, which is applied to improve the activity of the cementitious material thus increasing the mechanical strength. Due to this reason, the blast furnace slag powder and fly ash serve as the mineral admixtures. Additionally, the steel fibers can effectively the crack resistance of RPC, leading eventually to improving the mechanical strength and durability [30]. Therefore, the steel fibers are used in the RPC.

Magnesium phosphate cement RPC (MPC-RPC) may possess the advantages of fast hardening, early strength, excellent later mechanical strength and corrosion resistance [31,32]. This kind of material may act as a perfect cement-based material applied in replacing the cement concrete of anchorage zone of bridge expansion joint. The anchorage zone of bridge composed by MPC-RPC may be corroded when exposed to the chloride corrosion environment. Ultrasonic testing and electrical parameter methods are the nondestructive testing methods, which may be well used for reflecting the corrosion resistance of reinforced MPC-RPC. However, little attention about the corrosion resistance of reinforced MPC-RPC under the chloride corrosion environment has been reported.

In this paper, the working performance, the flexural strength, the compressive strengths and the electrical resistance of magnesium phosphate cement RPC are investigated. Moreover, the corresponding corrosion resistance of inner steel bars are researched. F-T cycles and D-W alternations with 3% NaCl are provided as the erosive environment. This study will develop new rapid hardening repair concrete, which may be well-applied in the marine and civil engineering environment.

## 2. Experimental

### 2.1. Raw Materials

The potassium dihydrogen phosphate manufactured by Shandong jinyida Chemical Co., Ltd., Jinan, China is used for making the magnesium phosphate cement (MPC). The purity and density of potassium dihydrogen phosphate are 98.5% and 2.238 g/cm^3^, respectively. Another power material, pyrophoric magnesium oxide with the density of 3.58 g/cm³ and purity of 99.1% (Xi’an Haotian Bioengineering Co., Ltd., Xi’an, China) is added. Blast furnace slag powder (BFS) and fly ash (FA) are provided by Huixin mining processing plant, Shijiazhuang, China. BFS shows the density of 2.9 g/cm^3^ and the specific surface area of 436 m^2^/g, and the thermogravimetric loss rate of 1.7%. The SiO_2_ content of quartz sand is 99%, which is composed of three particle sizes (0.71 mm~1 mm, 0.35 mm~0.59 mm and 0.15 mm~0.297 mm), and the proportions of the three particle sizes are 1:1.5:0.8, respectively. The water reducer used is polycarboxylic acid high-performance water reducer (SP) produced by Henan Pingdingshan admixture Co., Ltd., Pingdingshan, China. The water reduction rate can reach 40%. The dosage of water reducer is unified as 1.33% of the total mass of cementitious materials (the sum of the mass of potassium dihydrogen phosphate, magnesium oxide, BFS and FA), and the water binder ratio of MPC-RPC is 0.15. Table 1 and Table 2 show the cumulative passing rate and chemical composition of raw materials, respectively. The particle passing percentage of raw materials is obtained by screening experiment. The measuring process is carried out by the manufactory. Copper plated steel fibers show the density of 7.85 g/cm^3^, the tensile strength of 3125 MPa, the diameters of 0.10 mm~0.25 mm and the length of 10 mm~20 mm showing the average diameter and length of 0.21 mm and 15 mm, respectively.

### 2.2. Sample Preparation

Table 3 is the mix proportion of MPC-RPC per unit volume, which is used for making the MPC-RPC. The percentages of admixtures are obtained from prior studies [9,17,19], which are based on the maximum density theory. In addition, it is also convenient for comparison with previous studies. The specimens are manufactured by the following steps.

Firstly, the weighed dried raw materials (MgO, MgCl_2_, Borax, K_2_HPO_4_, FA and BFS) are added to the Hobart A200C mixer and combined at the stirring speed of (107 ± 5) rpm for 2 min. During the mixing, the steel fibers are scattered into the mixing pot in batches and combined at the mixing speed of (198 ± 5) rpm for another 1 min, finally the mixed liquids with water and water reducer are added to the mixing pot and 5 min stirring with the mixing speed of (361 ± 5) rpm provided for stirring the MPC-RPC mixture. The fresh MPC-RPC is used to measure the slump flow and setting time after the mixing is completed. NLD-3 electric jumping table cement mortar fluidity tester is used for the measurement of slump flow. ZKS-100 mortar setting time tester is used for the measurement of the setting of MPC-RPC. All measuring processes are referred in the published papers and the Chinese standards GB/T2419-2005 and JGJ/T 70-2009 [33,34]. After the above testing, the fresh MPC-RPC is poured into molds with sizes of 40 × 40 × 160 mm^3^ and 50 × 50 × 50 mm^3^ to form specimens.

### 2.3. Measurement Methods

#### 2.3.1. Mechanical Strengths

**Figure 1:**
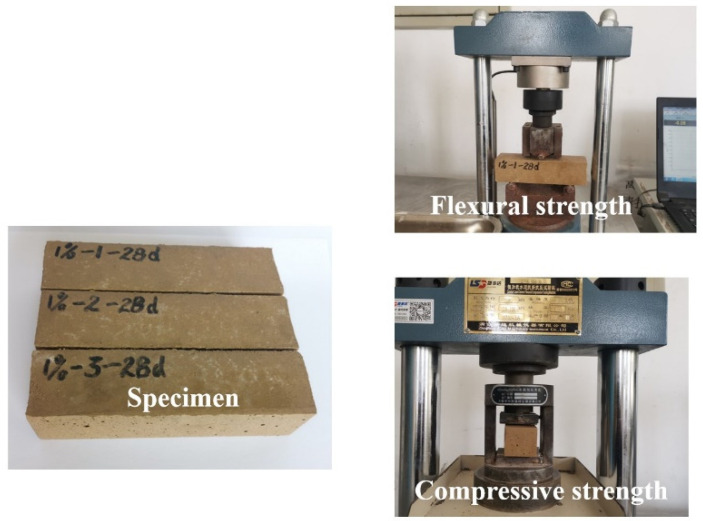
The process of measuring mechanical strength.

YAW-300E cement mortar compressive and flexural machine is used to measure the flexural and compressive strengths of specimens with size of 40 × 40 × 160 mm^3^. After the specimens are cured in the environment of 20 °C and relative humility of 99% for the needed curing ages, the specimens are moved to the bending fixture with the flat and smooth surface of test piece on the fixture. Then, load rate with 0.05 kN/s is provided for the flexural strength. After the flexural specimen is broken, two fault blocks are moved to the compressive fixture and the load with the loading rate of 2.4 kN/s is used [35]. The measuring process of mechanical strengths is shown in Figure 1.

#### 2.3.2. Ultrasonic Velocity Test

**Figure 2:**
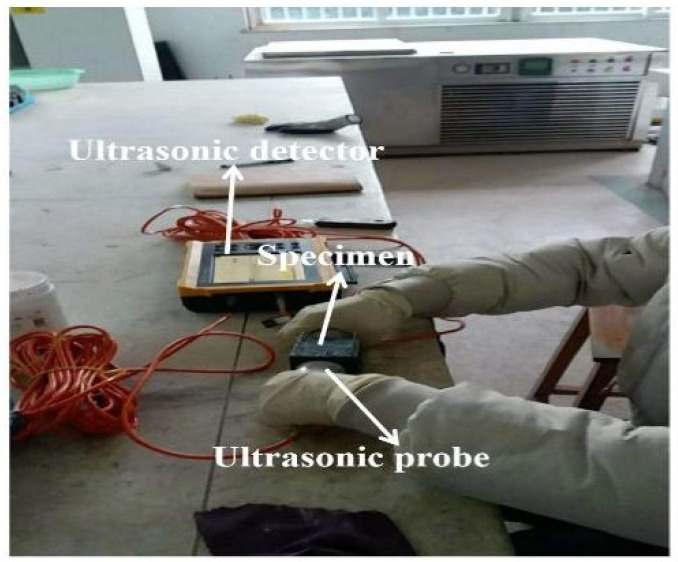
The ultrasonic velocity test process.

Specimens with size of 50 × 50 × 50 mm^3^ are applied in the determination of ultrasonic velocity. The Jinghong CJ-10 intelligent nonmetal ultrasonic detector manufactured by Cangzhou Jinghong Engineering Instrument Co., Ltd., Cangzhou, China is used for the measurement of ultrasonic velocity. The vaseline is coupled with the surface of the specimens before testing. Figure 2 shows the measurement of the ultrasonic velocity.

#### 2.3.3. AC Electrical Parameters Test

**Figure 3:**
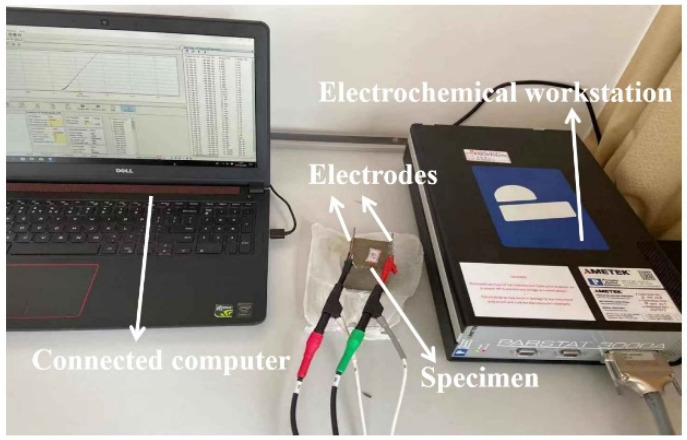
The measurement of AC electrical parameters.

The electrical parameters of specimens are tested by TH2810D LCR digital electric bridge (AC electrical resistance measurement) and PARSTAT 3000A electrochemical workstation (Determination of AC impedance spectrum). The testing frequency, the voltage and the sampling frequency of TH2810D are 10^4^ Hz, 1 V and 10 Hz. The testing frequency and voltage of PARSTAT 3000A electrochemical workstation are 10^5^ Hz~1 Hz and −10 mV~10 mV. Two pieces of 316 L stainless steel mesh with aperture’s diameter of 4 mm serve as two electrodes. The distance between two electrodes is 40 mm. The measurement of the electrical parameters are shown in Figure 3. The experimental details are described in Wang’s paper [36,37].

#### 2.3.4. Corrosion of Steel Bars Inner MPC-RPC under NaCl Eroded Environment

**Figure 4:**
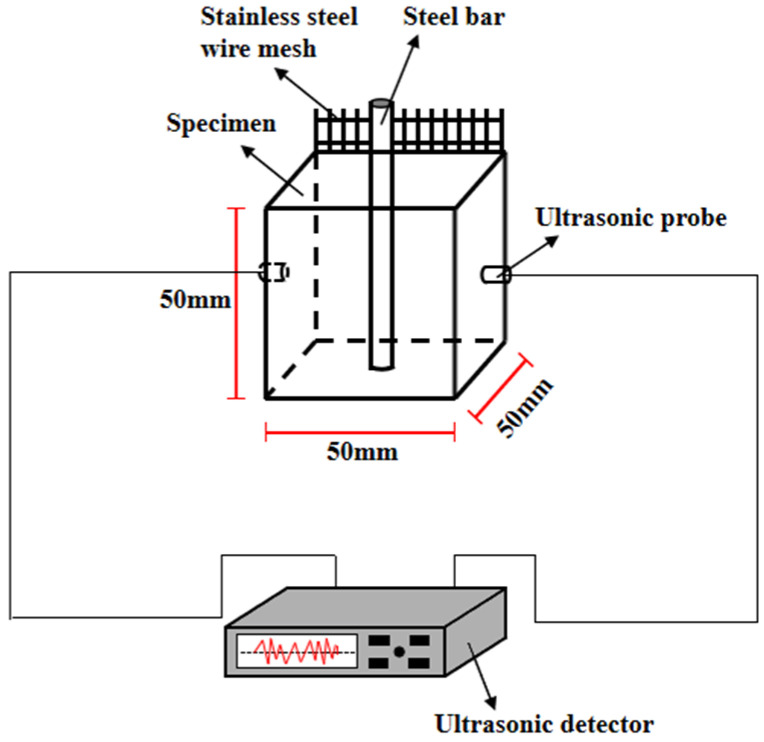
The ultrasonic velocity’s measurement.

**Figure 5:**
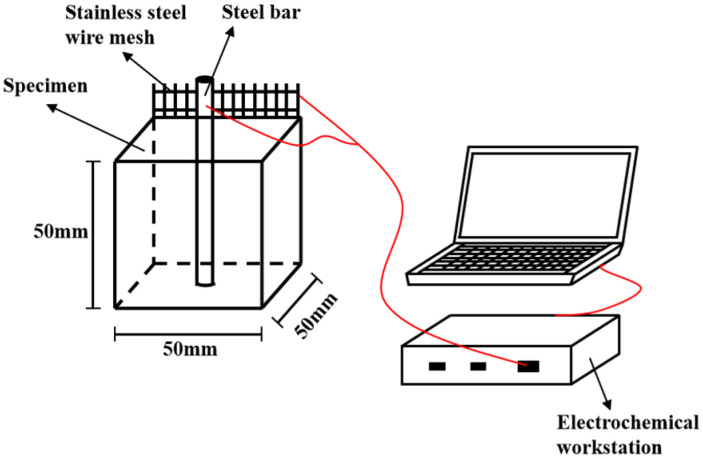
Measurement of AC electrical parameters.

The steel bars are embedded the center position of the MPC-RPC. The specimens are under the NaCl F-T cycles’ environment with the NaCl concentration of 3%. Fully automatic control concrete rapid freezing and thawing test box with the temperature range of −25 °C~50 °C and unit operating power of 5.5 kW is used for the freeze-thaw experiment. 24 days’ standard curing condition is provided for the specimens. After this, some specimens are immersed in the solution containing 3% NaCl for 4 days and are moved to the rapid freezing and thawing test box with the working temperature of −15 °C~8 °C. Some other specimens are used for the experiment of D-W alternations of NaCl solution. Firstly, the specimens are immersed in the NaCl solution for 10 h, then the specimens are dried in the DHG series vertical 300 °C blast drying oven with the temperature of 60 °C for 36 h. Finally, the specimens are moved from the drying oven and cooled in the temperature of 20 °C and relative humility of 40% for 2 h, until the next D-W cycle. The mass loss rate, the ultrasonic velocity, the electrical resistance and the AC impedance spectrum of specimens during the corrosion process are obtained. The measuring methods are the same as RPC specimens without steel fibers. A 316 L stainless steel mesh serves as an electrode, meanwhile, the embedded steel bar is used as another electrode. The experimental measurements of corrosion of steel bars inner RPC are exhibited in Figure 4 and Figure 5. In this study, 3 specimens are used for the measurement of mechanical strength, and 6 specimens are applied in the test of electrical parameters and ultrasonic velocity.

## 3. Results and Discussions

### 3.1. The Workability of MPC-RPC

**Figure 6:**
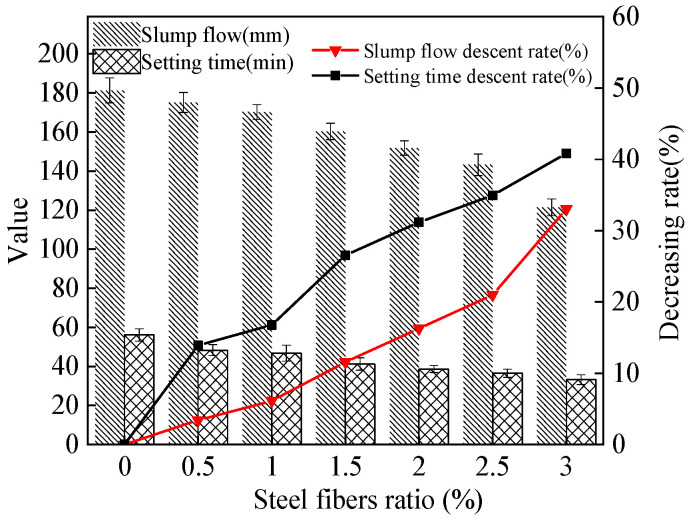
The working performance of MPC-RPC.

The slump flow and setting time of the MPC-RPC are shown in Figure 6. Figure 6 shows that when the ratio of steel fibers increases, the slump flow and the setting time decrease. This is mainly because the steel fibers’ networks can prevent the flow of fresh MPC-RPC paste [38]. Therefore, the slump flow of fresh MPC-RPC slurry will be decreased due to the addition of steel fibers. Moreover, as depicted in Figure 6, the setting time of MPC-RPC decreases with an increase in steel fibers ratio as the steel fibers may absorb some free water, leading to decreasing the setting time of MPC-RPC. Moreover, the addition of steel fiber will affect the physical state of MPC-RPC, thus decreasing the setting time of MPC-RPC [39]. The values of error bars are lower than 0.096, which ensures the experiment’s accuracy. The setting time of MPC-RPC ranges from 33.2 min to 56.1 min, which provides sufficient operation time for construction. Meanwhile, the slump flow of fresh MPC-RPC is 121.4 mm~181.3 mm, which ensures the sufficient fluidity during pouring.

### 3.2. The Mechanical Strengths of MPC-RPC

**Figure 7:**
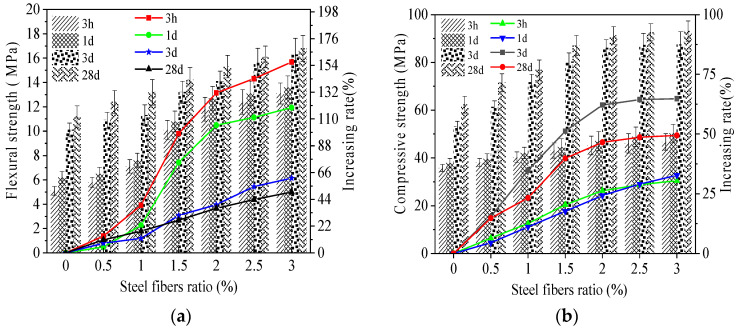
The mechanical strengths of MPC-RPC. (**a**) The flexural strength. (**b**) The compressive strength.

As reported in References [3,9], the curing ages of 3 h, 1 day, 3 days and 28 days are usually used for reflecting the mechanical strength of magnesium phosphate cement-based material. In order to facilitate comparison with previous studies, the curing ages of 3 h, 1 day, 3 days and 28 days are selected. The flexural and compressive strengths of MPC-RPC are depicted in Figure 7. It can be observed in Figure 7, the flexural and compressive strengths increase with the increasing steel fibers ratio and the curing age. The flexural strength of MPC-RPC cured for 3 h increases by 156.9%, when the steel fibers ratio varies from 0% to 3%. While, when the curing ages are 1 day, 3 days and 28 days, the increasing rates are 119.4%, 61.4% and 50%, respectively. Moreover, the increasing rates of compressive strength by steel fibers of specimens cured for 3 h, 1 day, 3 days and 28 days are 0~30.4%, 0~32.7%, 0~64.8% and 0~50%, respectively. When the curing age ranges from 3 h to 1 d, the increasing rate of flexural strength of MPC-RPC is 3.8~21.5%. Meanwhile, when the curing age ranges from 3 h to 28 days, the increasing rate of flexural strength of MPC-RPC is 28.2~119.6%. Additionally, the maximum increasing rate of compressive strength by curing age is 102.1%. Meanwhile, the maximum increasing rate of compressive strength by the steel fibers ratio is 49.4%. This is attributed to the reason that the magnesium oxide will react with potassium dihydrogen phosphate to form hydrated magnesium phosphate rapidly [40,41,42], besides the mechanical strengths of MPC-RPC at low curing age is enough high. The addition of steel fibers can bridge cracks inner MPC-RPC, thus improving the mechanical strengths, especially the flexural strength. The values of error bars are lower than 0.073, indicating the accuracy of experimental results. Compared with the RPC prepared with sulphoaluminate cement, the flexural strength of MPC-RPC is reduced by 13.2~25.4%, and the compressive strength is increased by 10.6~31.3% [43]. The flexural strength and compressive strength of MPC-RPC before 28 days are 25% higher than those of RPC prepared with ordinary Portland cement. Meanwhile, when the curing age is 28 days, the mechanical strengths of MPC-RPC are lower than that of RPC with Ordinary Portland cement [44].

### 3.3. The Ultrasonic Velocity of MPC-RPC

**Figure 8:**
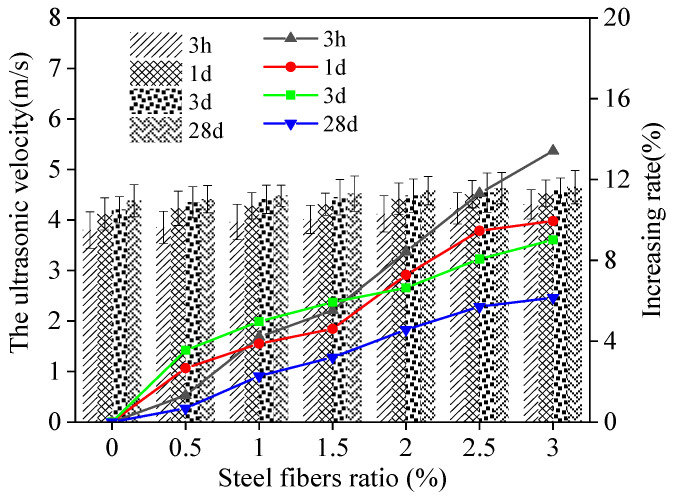
Ultrasonic velocity of MPC-RPC.

The ultrasonic velocity (*v*) of MPC-RPC after curing for 3 h, 1 day, 3 days and 28 days, is shown in Figure 8. It can be noticed in Figure 8, as the curing age increases and steel fibers are added, the ultrasonic velocity also increases. This is explained by the fact that when the curing age increases, the amount of hydrated magnesium phosphate increases and MPC-RPC become more compact, which increases the ultrasonic velocity [43]. Moreover, the increased dosages of steel fibers can improve the compactness of steel fibers’ networks, which results in increasing the ultrasonic velocity. In comparison to 80% of the specimens that are cured for 28 days, the ultrasonic velocity of specimens cured for 3 h is higher. Additionally, the ultrasonic velocity of blank specimens is higher than 81.7% of the specimens with 3.0% steel fibers. The flexural strength, the compressive strength and ultrasonic velocity of RPC cured for 3 h are higher than 45% of the MPC-RPC cured for 28 days. The flexural strength, the compressive strength and ultrasonic velocity of MPC-RPC cured for 7 days are higher than 85% of the specimens cured for 28 days. The values of error bars are all lower than 0.085, ensuring the precision of researching results. The ultrasonic velocity of MPC-RPC is 5.4~11.3% lower than that of RPC with Ordinary Portland cement [44,45]. The detailed data of Figure 6, Figure 7 and Figure 8 have been provided by the graphs be presented in the form of tables at the end of the article (Appendix A).

### 3.4. Electrical Resistivity of MPC-RPC

**Figure 9:**
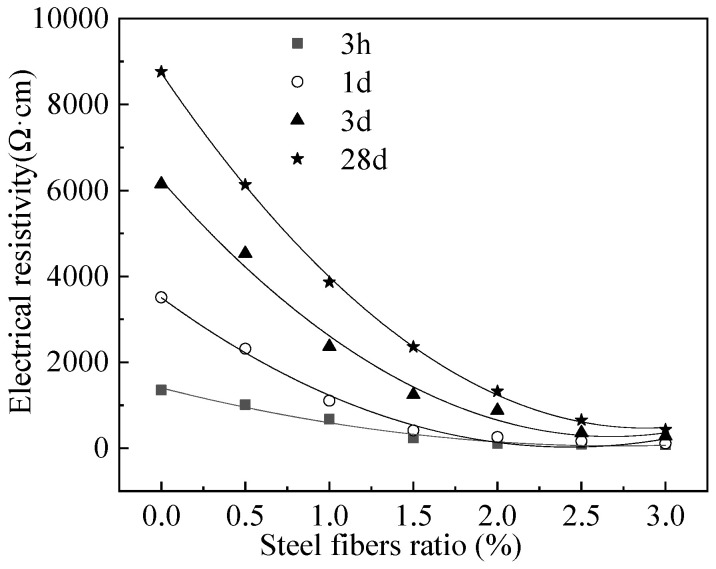
Electrical resistivity of MPC-RPC.

The electrical resistivity (*ρ*) of MPC-RPC is shown in Figure 9. As illustrated in Figure 9, the electrical resistivity of MPC-RPC decreases in the form of quadratic function. The fitting results of the relationship between *ρ* and *V* are listed in Table 4, and it can be seen that the fitting degree is higher than 0.99, which verifies the rationality of the fitted equation. When the volume ratio (*V*) of steel fibers increases from 0% to 1.5%, the electrical resistivity of MPC-RPC drops obviously. This is ascribed to the fact that the conductive fiber networks come into being, therefore, the electrical resistivity is very sensitive to the increase of steel fibers’ content [46]. While, when the steel fibers’ volume ratio is higher than 1.5%, the electrical resistivity of MPC-RPC tends to be stable, due to the complete conductive fibers’ network. Hence, the increasing steel fibers’ ratio has little influence on the conductive performance of MPC-RPC.

### 3.5. Corrosion Resistance of Steel Bars Inner MPC-RPC

**Figure 10:**
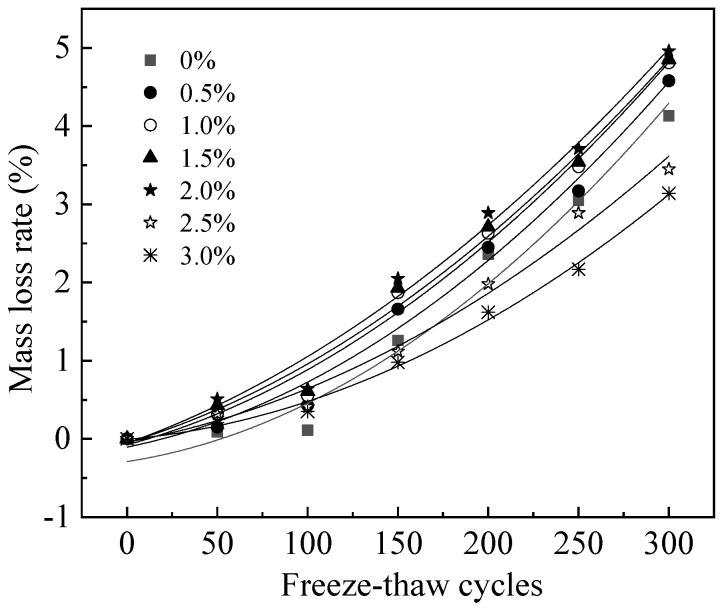
The mass loss rate of reinforced MPC-RPC during NaCl F-T cycles.

The mass loss rate (Δ*m*/*m*) of reinforced MPC-RPC during NaCl F-T cycles(*N*) is illustrated in Figure 10. The fitting results of the relationship between (Δ*m/m*) and the *N* are shown in Table 5. Figure 10 shows that as the number of NaCl F-T cycles (*N*) increases, so does the mass loss rate of reinforced MPC-RPC. This is explained by the fact that frozen-heave stress can make the surface on the cement concrete spall [47]. Moreover, the chloridion can corrode the steel bars and steel fibers inner MPC-RPC thus causing cracking of MPC-RPC and reducing the mass of MPC-RPC. When the steel fibers’ ratio varies from 0% to 2.0%, the mass loss increases up as the dosage of steel fibers is increased, due to the fact that the steel fibers enhance the loss of electronic capability. When the dosage of steel fibers is 2.0%~3.0%, the mass loss rate of reinforced MPC-RPC decreases with the increasing steel fibers’ ratio. This is explained by the ability of the steel fibers to bridge internal cracks in MPC-RPC, which decreases the mass loss of MPC-RPC [48].

**Figure 11:**
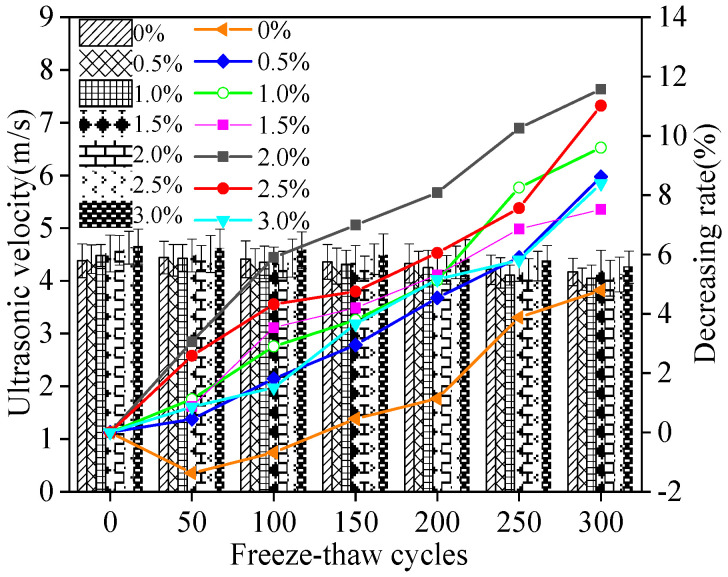
The ultrasonic velocity of MPC-RPC during NaCl F-T cycles.

The variation rate of ultrasonic velocity of MPC-RPC is illustrated in Figure 11. The ultrasonic velocity decreases with the increasing number of NaCl F-T cycles, as shown in Figure 11. This is explained by the fact that the NaCl F-T cycles can accelerate the MPC-RPC’s crack propagation which blocks the propagation process of ultrasound [49,50]. Additionally, the chloride ions will penetrate into MPC-RPC through the cracks developed by NaCl F-T cycles thereby corroding the passive film of reinforcement and steel fibers thus accelerating the following corrosion [28,29]. Consequently, the cracks increase and the ultrasonic velocity decreases. Moreover, as observed in Figure 11, in some conditions, the addition of steel fibers lead to decreasing the ultrasonic velocity, due to the increasing dosages of steel fibers can improve the electrical conduction of MPC-RPC, which accelerates the electrochemical corrosion of the inner reinforcement. On the other hand, the increasing dosage of steel fibers can limit the cracking of cracks, which increases the ultrasonic velocity [51]. The values of error bars are lower than 0.1, which exhibits accurate experimental results.

**Figure 12:**
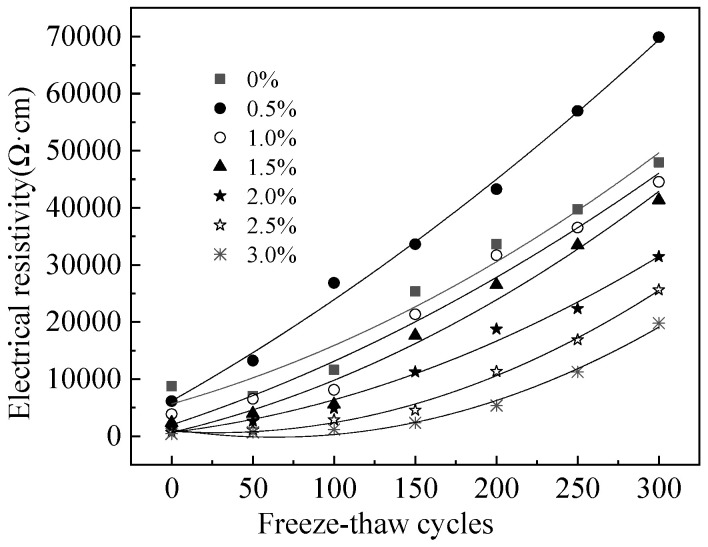
The electrical resistivity of MPC-RPC during NaCl F-T cycles.

Figure 12 presents the electrical resistivity of MPC-RPC. It is apparent from Figure 12 that the electrical resistivity increases with the increasing NaCl F-T cycles(N). The relationship between electrical resistivity and the number of F-T cycles is deduced as quadratic function. The fitting results are exhibited in Table 6. As shown in Table 6, the fitting degrees are higher than 0.99, thus proving the accuracy of the fitting equations. This is mainly because the NaCl F-T cycles can increase the F-T cracks which blocks the migration of conductive particles and increases the electrical resistivity of MPC-RPC [49,50]. Moreover, the NaCl F-T cycles can accelerate the corrosion of reinforcement and steel fibers. The rust inner MPC-RPC can prevent the electron transferring though reinforcement and steel fibers. Moreover, the rust can block the channel of pore solution, thus increasing the electrical resistivity [52,53]. Furthermore, the electrical resistivity of MPC-RPC is decreased by the increase in the dosage of steel fibers due to the improved steel fibers’ network.

**Figure 13:**
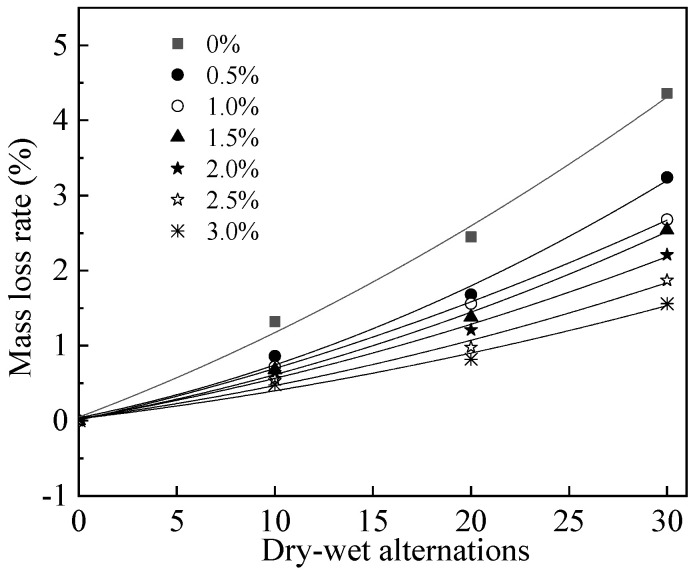
Mass loss rate of steel bars reinforced MPC-RPC during NaCl D-W alternations.

The mass loss rate of reinforced MPC-RPC during NaCl D-W alternations (*n*) is exhibited in Figure 13. As can be seen in Figure 13, the mass loss rates of all curves increase as a quadratic function with the NaCl D-W alternations. The fitting results are shown in Table 7. It can be found in Table 7, the fitting degrees are higher than 0.99, therefore, the fitting equation is reasonable. This is due to the fact that the NaCl D-W alternations can increase crystallization stress’ effect thus increasing the spalling of MPC-RPC and decreasing the following mass. Moreover, the NaCl D-W alternations lead to accelerating the corrosion of reinforcement and steel fibers, the rust by corrosion can induce the spalling on the surface of MPC-RPC, which decreases the mass [54,55].

**Figure 14:**
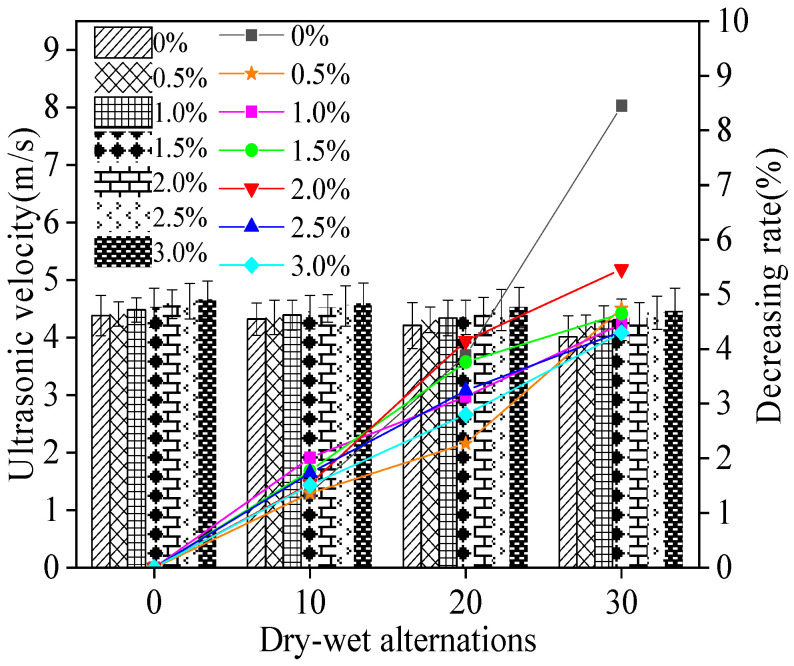
The ultrasonic velocity of MPC-RPC during NaCl D-W alternations.

Figure 14 demonstrates the ultrasonic velocity of MPC-RPC during NaCl D-W alternations. It can be noticed from Figure 14 that the ultrasonic velocity of MPC-RPC decreases with the increasing NaCl D-W alternations due to the increased inner cracks by NaCl D-W alternations. Moreover, the increased steel fibers’ dosages can form dense networks inner MPC-RPC, which increases the ultrasonic velocity of MPC-RPC.

**Figure 15:**
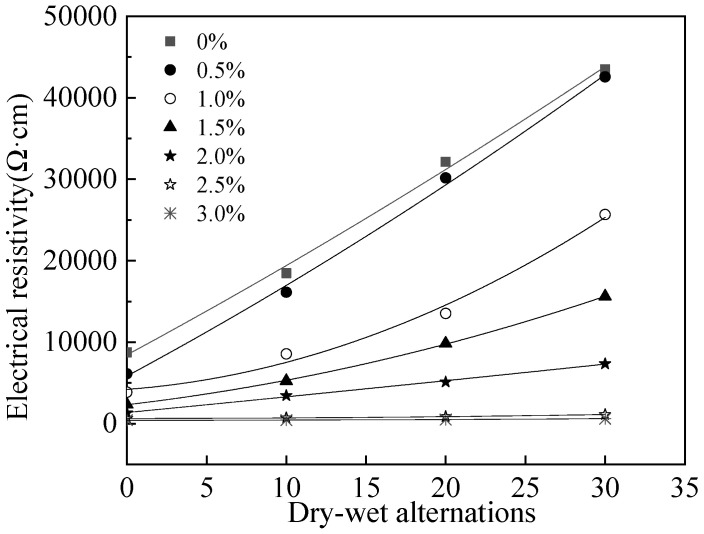
The electrical resistivity of MPC-RPC during NaCl D-W alternations.

Figure 15 shows the electrical resistivity of MPC-RPC during NaCl D-W alternations. As depicted in Figure 15, the electrical resistivity of MPC-RPC increases with the increasing number of NaCl D-W alternations. This is due to the fact that the NaCl D-W cracks induced by NaCl D-W alternations can reduce the transmission speed of conductive particles, therefore, the electrical resistivity of MPC-RPC is increased by the NaCl D-W alternations [55]. Table 8 illustrates the fitting results. As illustrated in Figure 15 and Table 8, the relationship between electrical resistivity of MPC-RPC and the number of NaCl D-W alternations fits well with quadratic function. Moreover, the corrosion degree of steel fibers and steel bars are increased by NaCl D-W alternations, resulting in a higher electrical resistivity of MPC-RPC [56,57]. Furthermore, the electrical resistivity of MPC-RPC is decreased with the increase in the increasing amount of steel fibers, due to the improved conductive networks by steel fibers.

**Figure 16:**
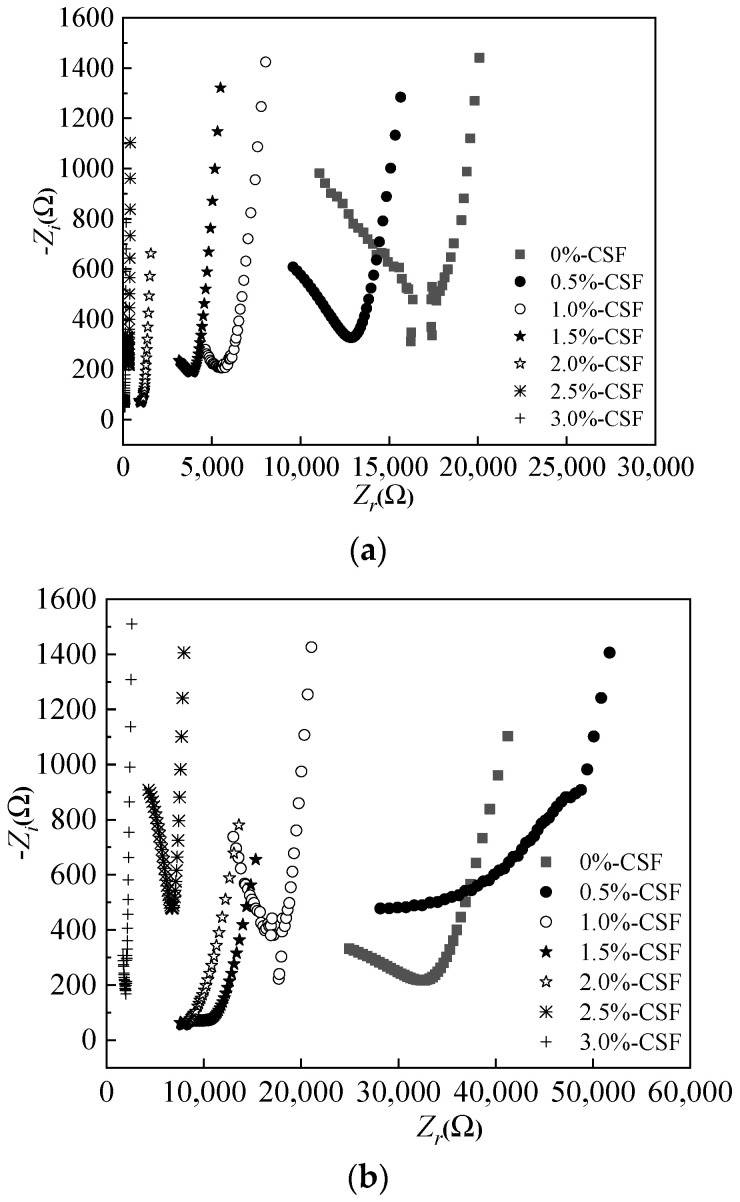

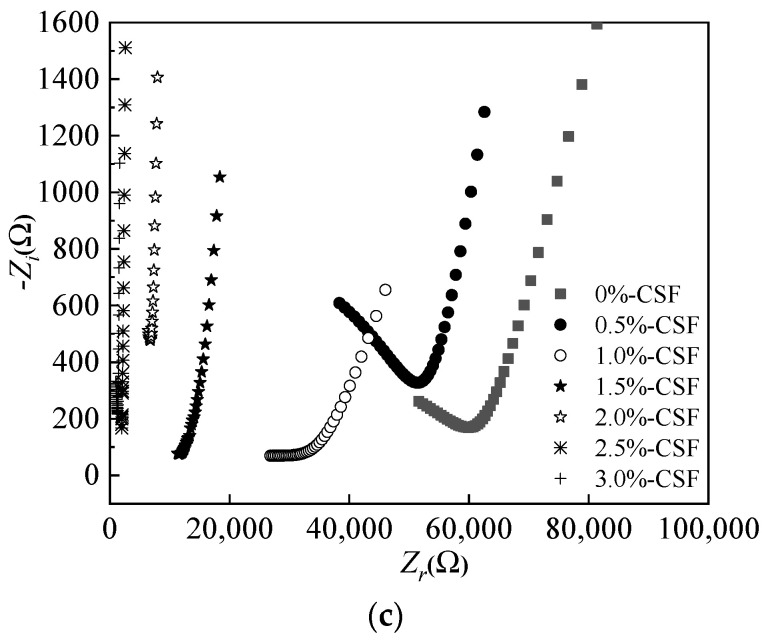
AC impedance spectrum curves of reinforced MPC-RPC. (**a**) Before corrosion. (**b**) After 300 NaCl F-T cycles. (**c**) After 30 NaCl D-W alternations.

Figure 16 depicts the AC impedance spectrum curves of reinforced MPC-RPC. The AC impedance spectrum curves consist of real part and imaginary part. The real part represents the electrical resistance. Meanwhile, the imaginary part refers to the electrical reactance. The imaginary parts of all curves firstly decrease and then increase with the increasing real part. As illustrated in Figure 16, the values of extreme point move from right to the left when the amount of steel fibers is increased, indicating enhanced electrical conduction. Moreover, it can be found in Figure 16, the NaCl F-T cycles and NaCl D-W alternations lead to increasing the real parts’ values of the extreme point. This is due to the increased electrical resistance by NaCl F-T cycles and NaCl D-W alternations, reflecting that the corrosion degree of reinforcement has been accelerated [55]. Furthermore, the increasing rate the values of extreme point by NaCl D-W alternations are higher than that by NaCl F-T cycles.

**Figure 17:**
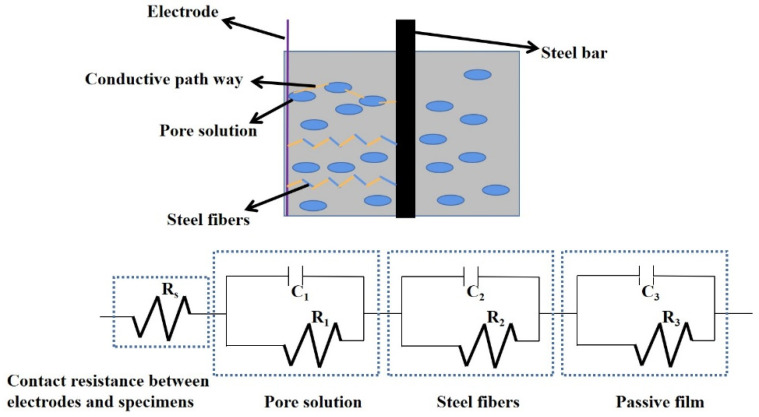
AC impedance spectrum curves.

Figure 17 shows the equivalent circuit diagram of reinforced MPC-RPC. The electric circuit of reinforced MPC-RPC is consisted of three parallel electrical components (the parallel electrical resistance and reactance of passive film, steel fibers and pore solution), as detailed in Figure 17. The corresponding Chi-squared is lower than 0.01, indicating the rationality of equivalent circuit diagram.

**Figure 18:**
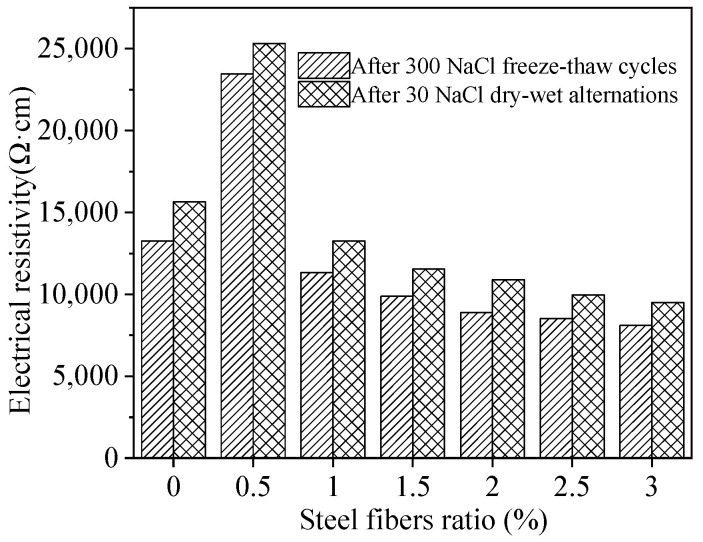
The electrical resistivity of the passive film through equivalent circuit diagram.

Figure 18 shows the electrical resistivity calculated by the equivalent circuit diagram of Figure 17. As can be seen in observed from Figure 18, the electrical resistivity of passive film increases when the addition of steel fibers increases from 0% to 0.5%, the electrical resistance increases with amount of steel fibers used, due to the increased electrochemical corrosion of inner steel bars [55]. However, when the dosages of steel fibers increase from 0.5% to 3.0%, the electrical resistivity of passive film decreases with the increasing steel fibers. This is attributed to the improving effect of steel fibers on the corrosion resistance of steel bars. Finally, it can be found that the electrical resistivity of the passive film of the specimens after 30 NaCl D-W alternations is higher than that after 300 NaCl F-T cycles. Therefore, the steel bars inner MPC-RPC corrode more seriously after 30 NaCl D-W alternations than that after 300 NaCl F-T cycles.

## 4. Conclusions

This paper aims to develop the rapid repairing cement-based material named MPC-RPC. The corrosion resistance of reinforced MPC-RPC exposed to the environment of NaCl F-T cycles and D-W alternations is systematically studied. The working performance and mechanical properties of MPC-RPC are investigated. The corresponding corrosion resistance of reinforced MPC-RPC exposed to the environment of is obtained. The conclusions are derived as follows.

The slump flow and the setting time of fresh MPC-RPC are decreased by an increase in the dose of steel fibers. The lowest slump flow and the setting time are 33.2 min and 121.4 mm.

The addition of steel fibers demonstrates positive effect on the flexural and compressive strengths of hardened MPC-RPC. The flexural strength, the compressive strength and ultrasonic velocity of RPC cured for 3 h are higher than 45% of the MPC-RPC cured for 28 d. The flexural strength, the compressive strength and ultrasonic velocity of MPC-RPC cured for 7 d are higher than 85% of the specimens cured for 28 d. The increasing rate of flexural strength by steel fibers of MPC-RPC cured for 3 h is 0%~156.9%, meanwhile, when the curing ages are 1 d, 3 d and 28 d, the increasing rates are 0%~119.4%, 0%~61.4% and 0%~50%, respectively. Additionally, the increasing rates of compressive strength by steel fibers of specimens cured for 3 h, 1 d, 3 d and 28 d are 0%~30.4%, 0%~32.7%, 0%~64.8% and 0%~50%, respectively. The maximum increasing rate by steel fibers is 18.3%.

When the reinforced MPC-RPC is exposed to the NaCl F-T cycles and NaCl D-W alternations, the mass loss rate and the electrical resistivity increase in the form with the numbers of NaCl F-T cycles and NaCl D-W alternations. The electrical mechanism can be explained by an equivalent circuit, which is tandem parallel electrical resistance and reactance of pore solution, steel fibers and passive film. As obtained from the results of the ultrasonic velocity, the mass loss rate, and the AC impedance spectrum, the steel fibers can improve the corrosion resistance of reinforced MPC-RPC, except 0.5% steel fibers. Moreover, the reinforced MPC-RPC corrodes more seriously exposed to NaCl D-W alternations than NaCl F-T cycles.

## Figures and Tables

**Table 1:** Particle passing percentage of raw materials (%).

Materials	0.3 μm	0.6 μm	1 μm	4 μm	8 μm	64 μm	360 μm
BFS	0.03	0.1	3.5	19.6	35.0	97.9	100.0
Quartz sand	0.0	0.0	0.0	0.0	0.03	20.0	100.0
FA	12.3	41.7	66.2	100.0	100.0	100.0	100.0

**Table 2:** The chemical composition of cement (%).

Materials	SiO_2_	Al_2_O_3_	Fe_x_O_y_	MgO	CaO	SO_3_	K_2_O	Na_2_O	Ti_2_O	Loss on Ignition
BFS	34.10	14.70	0.20	9.70	35.90	0.20	2.90	—	—	2.30
Quartz sand	99.60	—	0.40	—	—	—	—	—	—	—
FA	55.00	20.00	6.00	10.20	4.50	0.11	1.26	2.13	0.06	0.74

**Table 3:** The mix of RPC per unit volume (kg/m^3^).

Samples	Water	MgO	MgCl_2_	Borax	K_2_HPO_4_	FA	BFS	Quartz Sand	Steel Fibers
MPC-RPC-0	183.3	695.5	17.4	13.9	13.9	370.3	111.1	977.9	0
MPC-RPC-0.5	183.3	695.5	17.4	13.9	13.9	370.3	111.1	977.9	39.3
MPC-RPC-1.0	183.3	695.5	17.4	13.9	13.9	370.3	111.1	977.9	78.5
MPC-RPC-1.5	183.3	695.5	17.4	13.9	13.9	370.3	111.1	977.9	117.8
MPC-RPC-2.0	183.3	695.5	17.4	13.9	13.9	370.3	111.1	977.9	157.0
MPC-RPC-2.5	183.3	695.5	17.4	13.9	13.9	370.3	111.1	977.9	196.3
MPC-RPC-3.0	183.3	695.5	17.4	13.9	13.9	370.3	111.1	977.9	235.5

**Table 4:** The fitting results of the relationship between *ρ* and *V*.

Equation	Curing Age	*a*	*b*	*c*	R^2^
$\rho =aV^{2}+bV+c$	3 h	182.56	−992.64	1403.84	0.99
	1 day	589.15	−2863.59	3506.89	0.99
	3 days	832.44	−4457.68	6240.32	0.99
	28 days	998.57	−5745.31	8735.05	1.00

**Table 5:** The fitting results of the relationship between (Δ*m*/*m*) and the *N*.

Equation	Steel Fibers’ Ratio	*a*	*b*	*c*	R^2^
$\frac{\Delta m}{m}=aN^{2}+bN+c$	0%	3.90 × 10^−5^	3.56 × 10^−3^	−0.29	0.97
	0.5%	3.63 × 10^−5^	4.69 × 10^−3^	−0.11	0.99
	1.0%	3.35 × 10^−5^	6.26 × 10^−3^	−0.077	0.99
	1.5%	3.13 × 10^−5^	6.95 × 10^−3^	−0.049	0.99
	2.0%	2.86 × 10^−5^	8.22 × 10^−3^	−0.055	0.99
	2.5%	2.67 × 10^−5^	4.19 × 10^−3^	−0.042	0.99
	3.0%	2.78 × 10^−5^	2.05 × 10^−3^	−0.00595	1.00

**Table 6:** The fitting results of the relationship between *ρ* and the *N*.

Equation	Steel Fibers’ Ratio	*a*	*b*	*c*	R^2^
$\rho =aN^{2}+bN+c$	0%	0.22	80.2	5670	1.00
	0.5%	0.17	161	6190	0.99
	1.0%	0.18	93.7	2000	1.00
	1.5%	0.25	66.7	672	1.00
	2.0%	0.23	34.1	703	1.00
	2.5%	0.34	−18.6	896	0.99
	3.0%	0.34	−43.4	1210	0.99

**Table 7:** The fitting results of the relationship between (Δ*m*/*m*) and *n*.

Equation	Steel Fibers’ Ratio	*a*	*b*	*c*	R^2^
$\frac{\Delta m}{m}=an^{2}+bn+c$	0%	1.50 × 10^−3^	9.79 × 10^−2^	4.85 × 10^−2^	1.00
	0.5%	1.80 × 10^−3^	5.29 × 10^−2^	3.90 × 10^−2^	0.99
	1.0%	1.00 × 10^−3^	5.88 × 10^−2^	8.00 × 10^−3^	1.00
	1.5%	1.20 × 10^−3^	4.72 × 10^−2^	2.20 × 10^−2^	1.00
	2.0%	9.00 × 10^−3^	4.50 × 10^−2^	2.50 × 10^−2^	1.00
	2.5%	8.30 × 10^−4^	3.56 × 10^−2^	3.05 × 10^−2^	0.99
	3.0%	6.50 × 10^−4^	3.07 × 10^−2^	2.70 × 10^−2^	0.99

**Table 8:** The fitting results of the relationship between *ρ* and the *n*.

Equation	Steel Fibers’ Ratio	*a*	*b*	*c*	R^2^
$\rho =an^{2}+bn+c$	0%	4.15	1050	8450	1.00
	0.5%	6.01	1050	5850	1.00
	1.0%	0.186	147	4210	0.99
	1.5%	7.19	228	2340	1.00
	2.0%	0.277	190	1380	1.00
	2.5%	0.505	0.811	654	1.00
	3.0%	0.215	−0.14	434	1.00

## Appendix A

**Table A1:** The working performance of MPC-RPC.

Steel Fibers’ Ratio (%)	Slump Flow (mm)	Error Bars’ Values of Slump Flow	Setting Time (min)	Error Bars’ Values of Setting Time	Decreasing Rate of Slump Flow (%)	Decreasing Rate of Setting Time (%)
0.00	181.30	6.40	56.10	3.20	0.00	0.00
0.50	175.10	5.10	48.30	2.80	3.42	13.90
1.00	170.20	3.80	46.70	4.10	6.12	16.76
1.50	160.30	4.20	41.20	3.20	11.58	26.56
2.00	151.80	3.70	38.60	1.80	16.27	31.19
2.50	143.20	5.50	36.50	2.10	21.01	34.94
3.00	121.40	4.30	33.20	2.50	33.04	40.82

**Table A2:** The flexural strength strengths of MPC-RPC (MPa).

Steel Fibers’ Ratio (%)	3 h	Error Bars of 3 h	1 Day	Error Bars of 1 Day	3 Days	Error Bars of 3 Days	28 Days	Error Bars of 28 Days
0	5.1	0.36	6.2	0.48	10.1	0.57	11.2	0.88
0.5	5.8	0.41	6.5	0.53	10.9	0.65	12.4	0.94
1	7.1	0.55	7.6	0.59	11.3	0.86	13.2	1.05
1.5	10.1	0.79	10.8	0.86	13.2	0.91	14.2	1.03
2	11.8	0.98	12.7	0.98	14.1	0.84	15.3	0.92
2.5	12.4	1.01	13.1	1.01	15.6	1.21	16.1	0.89
3	13.1	0.86	13.6	0.95	16.3	1.33	16.8	1.06

**Table A3:** The compressive strength of MPC-RPC (MPa).

Steel Fibers’ Ratio (%)	3 h	Error Bars of 3 h	1 Day	Error Bars of 1 Day	3 Days	Error Bars of 3 Days	28 Days	Error Bars of 28 Days
0	35.8	1.44	37.9	1.92	53.1	2.28	62.3	3.52
0.5	38.1	1.64	39.6	2.12	61.3	2.6	71.5	3.76
1	40.3	2.2	42.1	2.36	71.6	3.44	76.8	4.2
1.5	43.1	3.16	44.6	3.44	80.3	3.64	87.1	4.12
2	45.2	3.92	47.1	3.92	86.1	3.36	91.3	3.68
2.5	46.1	4.04	48.9	4.04	87.3	4.84	92.6	3.56
3	46.7	3.44	50.3	3.8	87.5	5.32	93.1	4.24

**Table A4:** The ultrasonic velocity of MPC-RPC (m/s).

Steel Fibers’ Ratio (%)	3 h	Error Bars of 3 h	1 Day	Error Bars of 1 Day	3 Days	Error Bars of 3 Days	28 Days	Error Bars of 28 Days
0	3.8	0.36	4.12	0.32	4.201	0.26	4.38	0.32
0.5	3.85	0.32	4.23	0.34	4.35	0.31	4.41	0.27
1	3.96	0.35	4.28	0.26	4.41	0.28	4.48	0.21
1.5	4.01	0.28	4.31	0.22	4.45	0.35	4.52	0.35
2	4.12	0.36	4.42	0.31	4.48	0.33	4.58	0.28
2.5	4.23	0.31	4.51	0.27	4.54	0.39	4.63	0.31
3	4.31	0.29	4.53	0.26	4.58	0.25	4.65	0.33
